# Safe and curative modified two-stage operation for T4 esophageal cancer after definitive chemoradiotherapy: a case report

**DOI:** 10.1186/s40792-023-01692-x

**Published:** 2023-06-26

**Authors:** Tasuku Matsumoto, Kazuhiro Noma, Naoaki Maeda, Takuya Kato, Kazuya Moriwake, Kento Kawasaki, Masashi Hashimoto, Shunsuke Tanabe, Yasuhiro Shirakawa, Toshiyoshi Fujiwara

**Affiliations:** 1 Department of Gastroenterological Surgery, Okayama University Graduate School of Medicine, Dentistry and Pharmaceutical Sciences, 2-5-1 Shikata-Cho, Kita-Ku, Okayama, 700-8558 Japan; 2 Department of Surgery, Hiroshima City Hiroshima Citizens Hospital, Hiroshima, Japan

**Keywords:** T4 esophageal cancer, Chemoradiotherapy, Esophagectomy, Two-stage operation

## Abstract

**Background:**

The prognosis of esophageal cancer (EC) with organ invasion is extremely poor. In these cases, definitive chemoradiotherapy (CRT) followed by salvage surgery can be planned; however, the issue of high morbidity and mortality rates persists. Herein, we report the long-term survival of a patient with EC and T4 invasion who underwent a modified two-stage operation after definitive CRT.

**Case presentation:**

A 60-year-old male presented with type 2 upper thoracic EC with tracheal invasion. First, definitive CRT was performed, which resulted in tumor shrinkage and improvement in the tracheal invasion. However, an esophagotracheal fistula subsequently developed, and the patient was treated with fasting and antibiotics. Although the fistula recovered, severe esophageal stenoses made oral intake impossible. To improve quality of life and cure the EC, a modified two-stage operation was planned. In the first surgery, an esophageal bypass was performed using a gastric tube with cervical and abdominal lymph node dissections. After confirming improved nutritional status and absence of distant metastasis, the second surgery was performed with subtotal esophagectomy, mediastinal lymph node dissection, and tracheobronchial coverage of the fistula. The patient discharged without major complications after radical resection and has been recurrence-free for 5 years since the start of treatment.

**Conclusion:**

A standard curative strategy could be difficult for EC with T4 invasion due to differences in the invaded organs, presence of complications, and patient condition. Therefore, patient-tailored treatment plans are needed, including a modified two-stage operation.

## Background

Esophageal cancer (EC) is the sixth most fatal malignant cancer [1]. Owing to the anatomical characteristics of the esophagus and cancer’s frequent occurrence in the middle thoracic esophagus, esophageal squamous cell carcinoma (ESCC) occasionally invades adjacent essential organs, such as the trachea, bronchus, lungs, and descending aorta [2, 3]. Approximately 15% of patients with EC in Japan are clinically diagnosed with advanced tumors with organ invasion (i.e., T4 in the Union for International Cancer Control [UICC] classification). Because tracheobronchial invasion can cause fatal events, such as hemoptysis or asphyxia [4], the prognosis of patients with T4 EC is extremely poor.

Definitive chemoradiotherapy (CRT), which is defined as CRT with 50.4 Gy or higher dose, is one of the radical nonsurgical treatments for locally advanced ESCC, including T4 cases [5]. This has become the primary treatment for patients who are medically inoperable or refuse surgery [6]. Definitive CRT is recommended in older patients given their increased risk of postoperative complications and possibility of reduced postoperative activities of daily living [7]. However, recurrence rates after definitive CRT are estimated at 40–75%, even when clinical complete response (CR) is achieved [8–11]. Therefore, salvage surgery, which involves esophagectomy after definitive CRT, improves recurrence rates and overall survival (OS). Although salvage surgery enables long-term survival [12], high morbidity and mortality, along with decreased quality of life (QOL), present substantial challenges. Therefore, the indications for salvage surgery are challenging and controversial.

In patients with tracheobronchial invasion, an EC esophagobronchial fistula occasionally occurs (20–30% incidence) during or after CRT [5]. These fistulas can lead to severe pneumonia and extreme reduction in the patient’s QOL, making it impossible to continue CRT. Therefore, esophagobronchial fistulas are associated not only with decreased QOL but also worsening prognoses.

To overcome these challenges, we planned a modified two-stage operation. First, bypass reconstruction using a gastric tube was performed, followed by radical surgery with esophagectomy and lymph node dissection. Herein, we report the long-term survival of a patient with ESCC and T4 invasion who underwent definitive CRT followed by radical esophagectomy.

## Case presentation

**Fig. 1:**
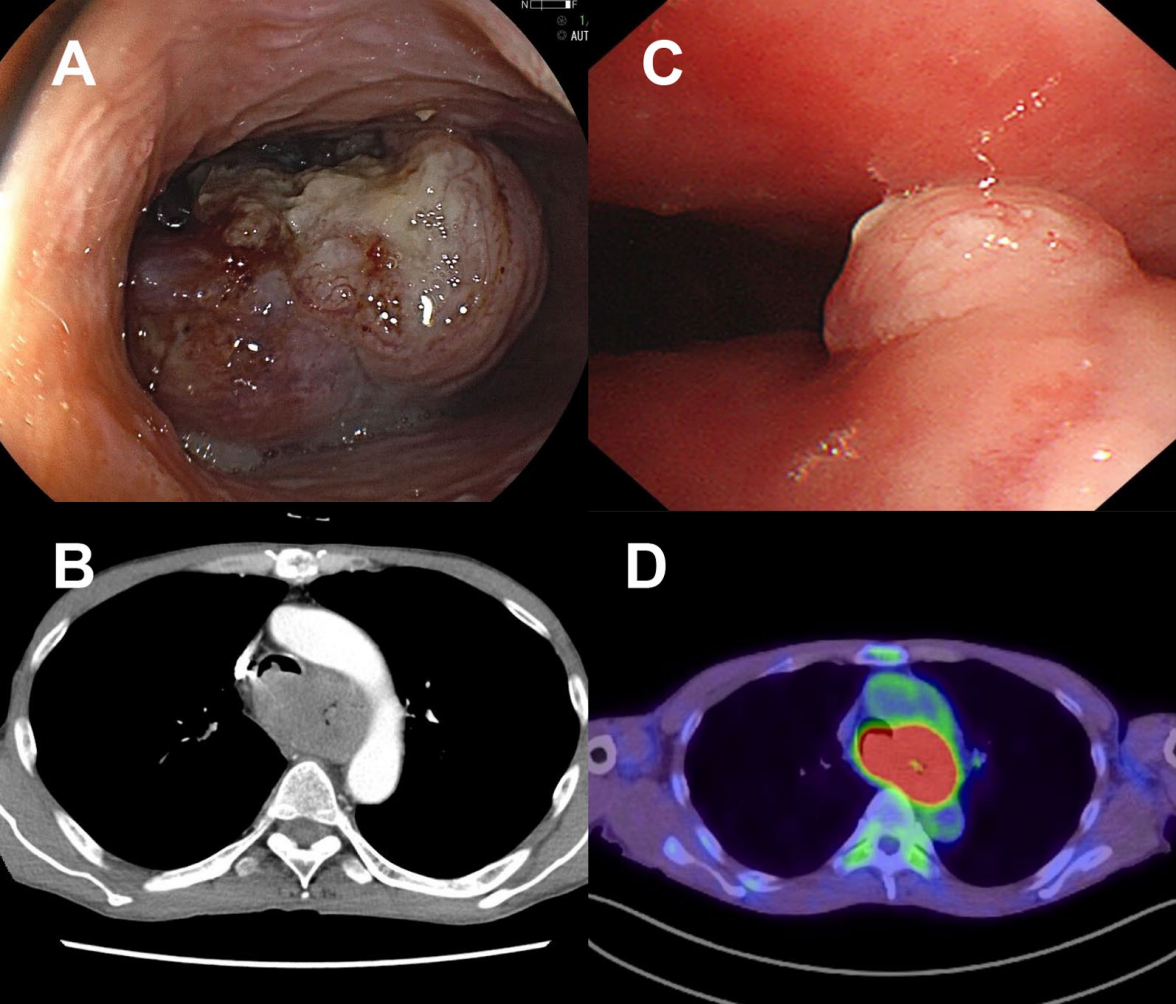
Initial medical examinations before treatment. **A** Esophagogastroduodenoscopy (EGD) image. A full circumferential type 3 tumor was detected in the upper thoracic esophagus. The scope was impassable. **B** Contrast-enhanced CT. A lumen-filling tumor with invasion of the tracheal bifurcation in the thoracic esophagus was shown. **C** Bronchoscopy. Partial tumor exposure near the tracheal bifurcation was indicated. **D** FDG PET-CT. There was an abnormal accumulation over a wide area of the thoracic esophagus, which was united with lymph nodes. There were no obvious distant metastases

A 60-year-old man presented with eating difficulties and dyspnea. Upper gastrointestinal endoscopy revealed two tumors in the cervical and middle thoracic esophagus, causing narrowing of the esophageal lumen, which was diagnosed as ESCC (Fig. 1A). Computed tomography (CT) revealed that an advanced tumor filled the lumen of the thoracic esophagus and partially compressed the trachea, with lymph node metastases in the bilateral paratracheal and the left supraclavicular lymph node. No findings were suggestive of distant metastasis (Fig. 1B). Furthermore, bronchoscopy revealed submucosal elevation of the tracheal mucosa and partial direct tumor invasion on the cephalic side of the tracheal bifurcation (Fig. 1C). ^18^F-fluorodeoxyglucose (FDG) positron emission tomography (PET)-CT showed extensive abnormal accumulation in the thoracic esophagus, partly lumped with lymph nodes. There were no obvious distant metastases (Fig. 1D). According to the UICC classification (8th ed.), the tumor was diagnosed as EC, UtMt, SCC cT4 (trachea) N2 M0 cStage IVa.

**Fig. 2:**
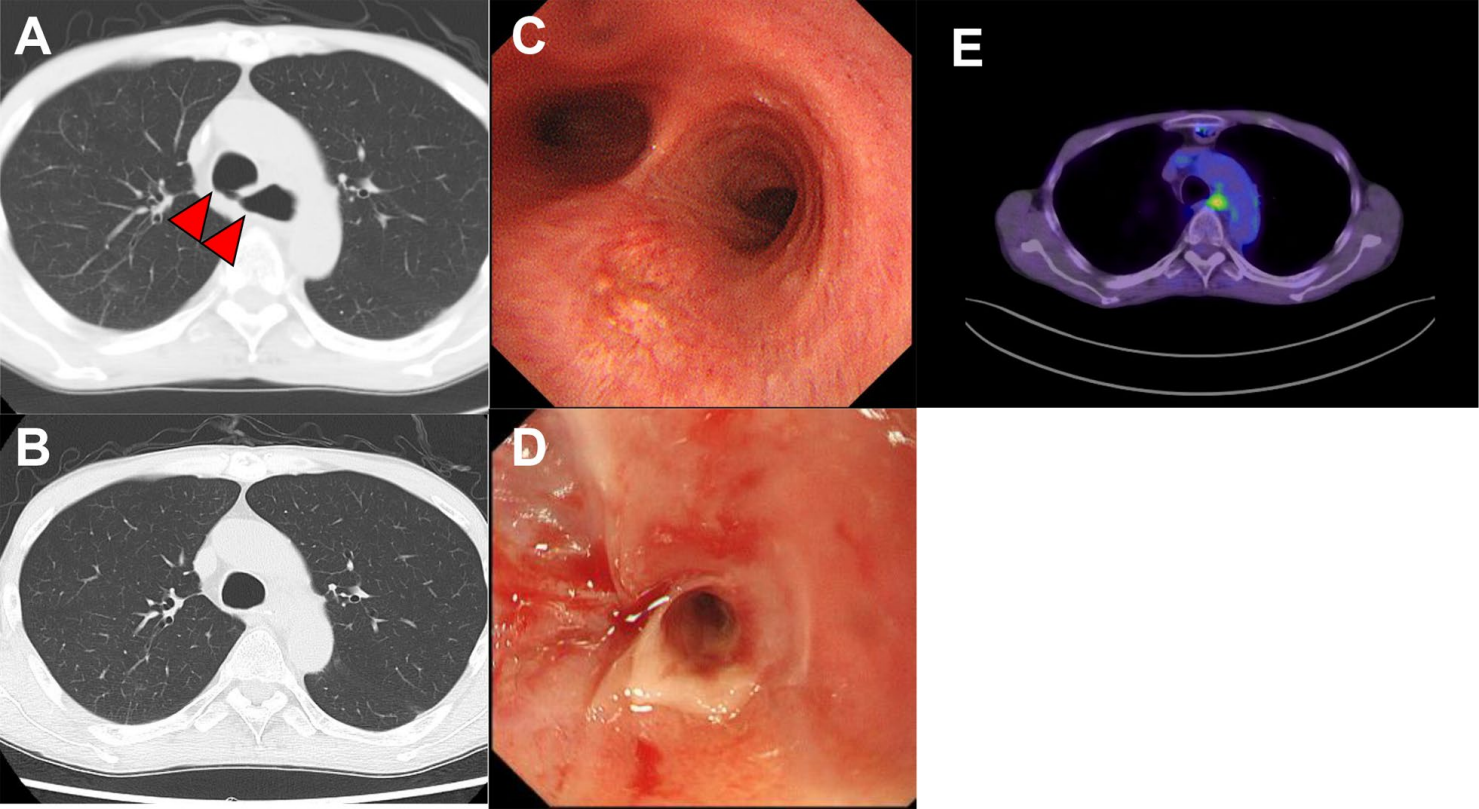
Examinations after definitive CRT. **A, B** CT images. Continuity between the trachea and esophageal lumen (arrowheads) was observed, suggesting esophagotracheal fistula. **B** CT after treatment for esophagotracheal fistula. The continuity disappeared. **C** Bronchoscopy. No fistula or tumor exposure were detected. **D** EGD image. Severe stenosis was developed, which prevented passage through the scope. **E** FDG-PET-CT after CRT. There was decreased accumulation of FDG in the thoracic esophagus, with no obvious distant metastases

To provide curative treatment, definitive CRT was performed; radiotherapy (1.86 Gy/day, 5 days/week, total 59.6 Gy) with docetaxel (75 mg/m^2^, 110 mg/body, day 1), cisplatin (70 mg/m^2^, 110 mg/body, day 1), and 5-fluorouracil (350 mg/m^2^, 1100 mg/body, days 1–5) continued for three courses. Clinical CR was achieved after definitive CRT; however, an esophagotracheal fistula was detected 2 months after this treatment (Fig. 2A). After 2 months of fasting and enteral nutrition using a gastrostomy, the esophagotracheal fistula was closed and cured (Fig. 2B, C). However, oral intake was impossible owing to severe stenosis after CRT (Fig. 2D). FDG PET-CT after CRT revealed decreased accumulation of FDG in the thoracic esophagus compared to the initial examination, without discernible evidence of distant metastases (Fig. 2E).

**Fig. 3:**
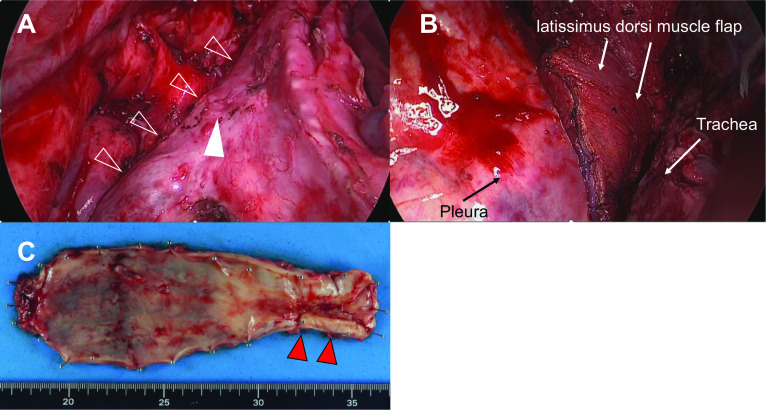
Perioperative findings. Intraoperative photo of second surgery. **A** Scarring of the fistula (filled arrowhead) in the tracheal membrane (open arrowhead). **B** Tracheobronchial fistula closure with the latissimus dorsi muscle flap. **C** Resected esophagus specimen. No EC was detected. The red arrow indicates severe stenosis

**Fig. 4:**
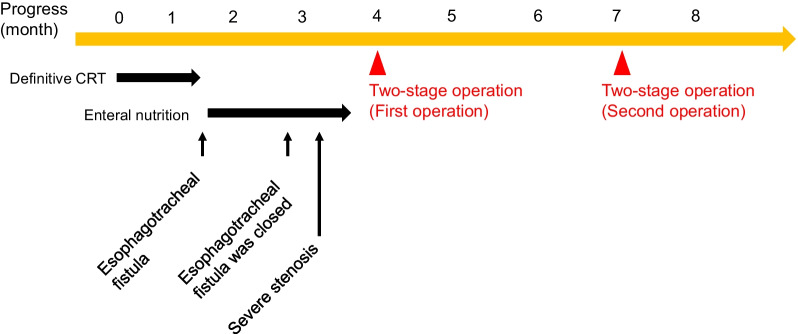
Treatment timeline. Four months after definitive CRT, a first-stage surgery was performed. Two months later, a second-stage surgery was performed

To allow oral intake and control tumor progression, a two-stage operation was planned. First, 4 months after CRT, an esophageal bypass using a gastric tube with cervical and abdominal lymph node dissection was performed because these areas were not irradiated with radiotherapy. Decompression was successfully accomplished by retrograde insertion of a 14Fr drainage tube into the residual esophagus, with subsequent extracorporeal guidance of the tube. Consequently, oral intake was possible on postoperative day 14 without complications. Following the operation, there was a notable enhancement in the patient's nutritional status, evidenced by a weight gain of 2 kg in comparison to the preoperative baseline. Moreover, the performance status (PS) exhibited substantial improvement, transitioning from 2 to 1 prior to surgery, thereby facilitating a successful discharge home without any episodes of pneumonia. One month after this surgery, no distant metastasis—except for the primary tumor in the remaining esophagus—was confirmed on follow-up FDG PET-CT. Additionally, a contrast-enhanced CT scan revealed a preoperative T stage diagnosis of T4 (trachea). Subsequent to CRT, the tumor exhibited substantial reduction in size, elimination of tracheal protrusion, and relatively distinct demarcation from the trachea. These favorable changes led to the determination of a curatively resectable tumor. Following a seven-month interval post-CRT, a second surgery was performed; a subtotal esophagectomy and mediastinal lymph node dissection. Esophagotracheal fistula scars were detected, and tracheobronchial fistula closure with a latissimus dorsi muscle flap was performed via right open thoracotomy (Fig. 3A, B). There were no serious complications except for mild pneumonia (Grade II, according to the Clavien–Dindo classification), and the patient was discharged on postoperative day 31. The pathological findings were diagnosed as CRT-pT1a N0 M0 and CRT-pStage IA (UICC 8th ed.; Fig. 3C). Figure 4 shows the treatment timeline. Lastly, no recurrence of EC was observed 5 years after the initiation of treatment.

## Discussion

In this case, CRT followed by radical surgery was performed to achieve radical resection of the locally advanced ESCC with tracheal invasion. Although an esophagotracheal fistula and esophageal stricture subsequently developed after CRT, a modified two-stage operation safely resolved these severe complications, improved the patient’s QOL, and achieved disease-free survival for 5 years.

This type of two-stage esophagectomy for ESCC was developed in the twentieth century to reduce surgical stress and critical complications; its advantage is a safe procedure that prevents the development of serious postoperative complications [13]. However, this method has some disadvantages, such as the necessity of double surgeries under general anesthesia and prolonged treatment period without oral intake. Currently, this strategy is only used in high-risk patients. For a two-stage operation, radical resection with lymph node dissection is usually planned first. However, in this case, reconstruction using a gastric tube was performed prior to radical resection to immediately avoid aspiration pneumonia due to esophageal stenosis, and improve performance status, nutritional status, and QOL. The patient’s nutritional status worsened due to continuous treatment for the esophageal stenosis and fistula; therefore, his general condition needed to be initially improved. Furthermore, a longer observation period is required to evaluate the emergence of metastatic lesions. Reducing the risk of distant metastasis recurrence was essential for local control by radical resection, which is highly invasive, to improve prognosis. Additionally, in the case of radical resection, if recurrence is detected before the second-stage surgery, the timing of reconstruction could be delayed, and poor QOL may be prolonged because cancer therapies are prioritized. In this case, the modified two-stage operation preceded by reconstruction had a favorable outcome. Thus, this approach was suggested as a feasible option in patients with poor general conditions or concerns about tumor control and distant metastasis.

Definitive CRT followed by esophagectomy provides similar OS proportions in patients with clinical stage III disease [14, 15]. In contrast, Nishimura et al. demonstrated that OS was improved in patients with stages I and II/III disease, but not in patients with unresectable locally advanced ESCC [16, 17]. Although this method remains controversial, some patients with T4 EC can achieve long-term survival by undergoing salvage surgery. Therefore, this method is promising for avoiding high complication rates associated with salvage surgeries.

Major treatments for esophagobronchial fistulas include stenting and bypassing it [18, 19]. Both are useful methods, but they have some disadvantages, including complications following implantation; these may cause life-threatening conditions such as fistula formation, migration, and bleeding [20, 21]. Esophageal bypass surgery has several postoperative complications associated with the procedure, including anastomotic leakage, stenosis, and recurrent nerve palsy [22]. In this case, the patient was treated with antibiotics, and a gastrostomy was performed for nutritional management, resulting in closure of the fistula. Therefore, conservative treatment may be an option for esophagobronchial fistulas in patients without severe pneumonia.

## Conclusion

Curative treatment with CRT followed by a modified two-stage operation can be safely performed prior to reconstruction using a gastric tube. T4 ECs are not uniformly associated with invaded organs, present symptoms, or patient condition; therefore, it is challenging to propose a standard treatment plan. Thus, developing patient-tailored treatment plans is necessary, and this strategy using a modified two-stage operation could be one way to achieve curative treatment for patients with T4 EC.

## Data Availability

All the data pertaining to this case report has been presented in the article.

## Abbreviations

CR: Complete response
CRT: Chemoradiotherapy
CT: Computed tomography
EC: Esophageal cancer
ESCC: Esophageal squamous cell carcinoma
QOL: Quality of life
UICC: Union for International Cancer Control
FDG PET-CT: 18F-fluorodeoxyglucose positron emission tomography-Computed tomography
